# Chest Pain Redefined: Atypical Presentation of Superior Mesenteric Artery Syndrome

**DOI:** 10.7759/cureus.56834

**Published:** 2024-03-24

**Authors:** Mohamed A Ebrahim, Eli A Zaher, Parth Patel, Bibek Adhikari, Muhammad Sohaib Alvi

**Affiliations:** 1 Internal Medicine, Ascension Saint Joseph Hospital, Chicago, USA

**Keywords:** aortomesenteric distance, ct, weight loss, aortomesenteric angle, sma

## Abstract

Superior mesenteric artery (SMA) syndrome is a rare cause of small bowel obstruction characterized by duodenal compression due to the narrowing of the SMA-aorta angle. We present a case of a 43-year-old male with postprandial chest pain, severe weight loss, and a narrowed aortomesenteric angle evident on computed tomography. Conservative management, including hydration, positioning, and weight gain, was initiated, leading to symptom resolution. SMA syndrome diagnosis requires clinical suspicion and radiological confirmation. Understanding this syndrome's varied presentations, diagnostic challenges, and therapeutic approaches is crucial for prompt management, especially when atypical symptoms like chest pain manifest, as seen in our case.

## Introduction

Superior mesenteric artery syndrome is a rare cause of small bowel obstruction. Loss of the mesenteric fat pad between the superior mesenteric artery (SMA) and aorta leads to the narrowing of the angle between the vessels, resulting in duodenal compression. Diagnosis is challenging due to non-specific symptoms and low incidence [1]. We describe a case of SMA syndrome presenting as postprandial chest pain.

## Case presentation

**Figure 1:**
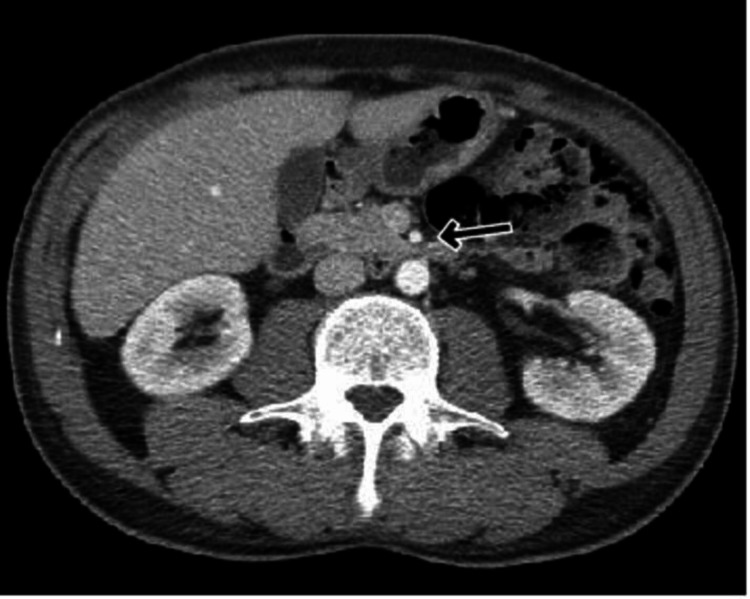
CT Abdomen Depicting an Aortomesenteric Distance of 6 mm Arrow pointing toward the distance between the aorta and superior mesenteric artery. The average normal aortomesenteric distance is 10-20 mm.

**Figure 2:**
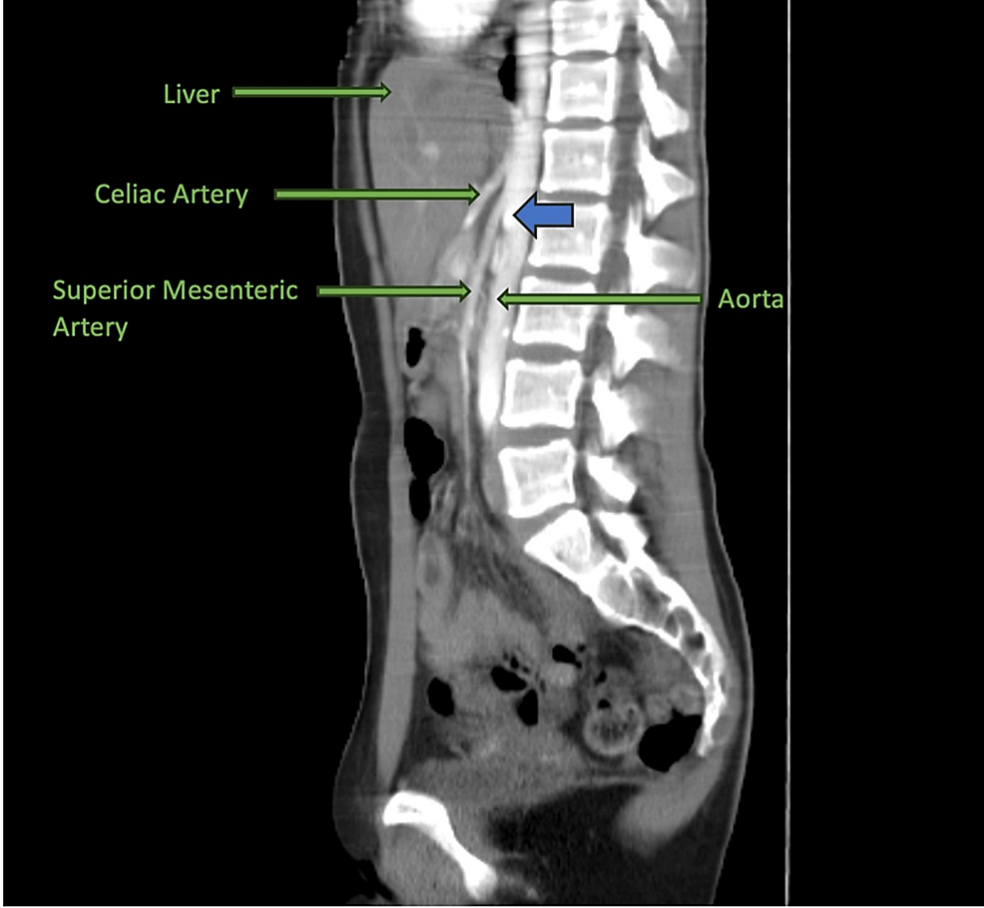
CT Abdomen Depicting an Aortomesenteric Angle of 19° Blue arrow pointing toward the angle between the aorta and superior mesenteric artery. The average normal aortomesenteric angle is 38°-56°.

A 43-year-old non-obese male with a history of gastroesophageal reflux disease (GERD) presented to the emergency department with complaints of chest pain starting the day prior. The pain was described as severe, post-prandial, with radiation to the back and was associated with nausea. He denied any use of drugs, smoking, or vomiting. He likewise endorsed an unintentional 25-pound weight loss in the preceding three months. No significant findings were noted on physical examination. Cardiac work-up and blood work were both unremarkable. Computed tomography angiography of the abdomen demonstrated a reduced aortomesenteric angle and a reduced aortomesenteric distance at the level of the duodenum (Figures 1-2).

General surgery and gastroenterology were thus consulted and recommended conservative management including intravenous hydration, lateral decubitus positioning, and weight gain. Following adequate tolerance to the general diet and lack of further symptoms, he was discharged home in good health with instructions to follow up with gastroenterology as an outpatient.

## Discussion

SMA syndrome, also known as Wilkie's syndrome, is a rare condition leading to intestinal obstruction [1,2]. It was initially identified by Austrian professor Carl Von Rokitansky in 1861, with Wilkie providing a comprehensive explanation of its pathophysiology in 1927, highlighting the association with a narrowed aortomesenteric angle and reduced distance [3]. The frequency of SMA syndrome in the general populace has been approximated to range from 0.013% to 0.78% according to radiographic investigations [1]. However, the precise prevalence of the condition remains uncertain, influenced by potential under- or over-diagnosis in clinical settings as well as the individual health status of each patient [4]. The third segment, or the transverse segment, of the duodenum takes a posterior-inferior path in relation to the point where the SMA originates. Normally, the angle between the abdominal aorta and SMA is around 45° (range: 38°-56°), with an aortomesenteric distance of 10-20 mm [5]. However, rapid loss of retroperitoneal fat can decrease this angle, potentially causing SMA syndrome, also referred to as aortomesenteric artery compression or duodenal vascular compression [6].

Factors that increase the likelihood of this syndrome encompass extended periods of bed rest in the supine position, swift weight reduction, individuals with a slender physique, and the use of body casts for correcting spinal trauma such as scoliosis, states of severe malnutrition, and various conditions leading to malabsorption. Additionally, anatomical factors like a short ligament of Treitz or an unusually low origin of the SMA can also contribute to SMA syndrome, particularly in children [7]. Treatment methods for scoliosis, such as surgery and casting, as well as scoliosis itself, are widely recognized as contributors to SMA syndrome. The elongation of the spine during scoliosis surgery is believed to be the fundamental mechanism behind this phenomenon. Additionally, intestinal surgeries like ileal pouch-anal anastomosis and colectomy are established factors, as they decrease the SMA-aortic angle by exerting tension on the mesentery [4].

Diagnosing SMA syndrome relies on a combination of clinical evaluation, suspicion, and radiological examinations. While barium studies may reveal non-specific findings, they often show dilation of the first and second segments of the duodenum followed by a sudden constriction at the third segment. Relief of obstruction can be observed when the patient is repositioned into a prone or left lateral decubitus position, allowing contrast material to flow freely into the small bowel beyond the SMA [8]. Computed tomography with contrast (CECT) presents similar findings of bowel obstruction, and reconstructed CT images offer an additional advantage by enabling measurement of the aortomesenteric angle and distance. As a result, CECT has become an essential component of the diagnostic process [9].

Typically, initial treatment involves conservative measures like postural changes or suction via a nasogastric tube to decompress the dilated stomach and duodenum. Positioning the patient in the left lateral or sitting position is often effective, although optimal positioning varies due to individual differences in SMA position and movement. Intravenous metoclopramide can aid in gastrointestinal motility enhancement alongside gastric tube suction. Following decompression, weight gain to increase adipose tissue between the SMA and aorta is highly advisable. Nasogastric feeding is effective, with jejunal tube feeding considered more ideal, and endoscopic assistance may be necessary [1,10]. Total parenteral nutrition serves as a viable option if intestinal feedings are unfeasible, contributing to the restoration of adipose tissue and increasing the SMA's origin angle. Surgical therapy is an option if conservative treatments fail, especially in elderly patients with a history of multiple abdominal surgeries, immobility, or longstanding SMA syndrome [11].

## Conclusions

Diagnosing SMA syndrome requires a high level of suspicion and careful correlation between clinical observations and radiological findings. Documenting such rare cases and their imaging characteristics can aid in determining the true incidence of the condition. In our case, the patient's clinical symptoms, although atypically presenting with chest pain, medical history, and radiological results guided us to the diagnosis of SMA syndrome. Despite its rarity, healthcare providers should consider the possibility of SMA syndrome when persistent symptoms, clinical suspicion, and imaging evidence are present.
